# Dual echo positive contrast bSSFP for real-time visualization of passive devices duringmagnetic resonance guided cardiovascular catheterization

**DOI:** 10.1186/s12968-014-0088-7

**Published:** 2014-10-28

**Authors:** Adrienne E Campbell-Washburn, Toby Rogers, Hui Xue, Michael S Hansen, Robert J Lederman, Anthony Z Faranesh

**Affiliations:** Cardiovascular and Pulmonary Branch, Division of Intramural Research, National Heart, Lung and Blood Institute, National Institutes of Health, Bethesda, MD 20892 USA

**Keywords:** Interventional MR, Cardiovascular magnetic resonance, Positive contrast, White marker, Guidewire, Real-time, Variable flip angle

## Abstract

**Background:**

Cardiovascular magnetic resonance (CMR) guided cardiovascular catheterizations can potentially reduce ionizing radiation exposure and enable new interventions. Commercially available paramagnetic X-Ray devices create a small signal void in CMR images, which is ambiguous and insufficient to guide catheterization procedures. This work aims to improve real-time CMR of off-the-shelf X-Ray devices by developing a real-time positive contrast sequence with color overlay of the device onto anatomy.

**Methods:**

A dual-echo bSSFP sequence was used to generate both a dephased positive contrast image and bSSFP image simultaneously. A variable flip angle scheme was implemented to reduce the specific absorption rate (SAR) and hence device heating. Image processing was used to isolate the device from background signal, and the device was overlaid in color on the anatomy, mimicking active device visualization. Proof-of-concept experiments were performed using a commercially available nitinol guidewire for left heart catheterization in Yorkshire swine.

**Results:**

The dual echo pulse sequence generated a temporal resolution of 175 ms (5.7 frames/second) with GRAPPA acceleration factor 4. Image processing was performed in real-time and color overlay of the device on the anatomy was displayed to the operator with no latency. The color overlay accurately depicted the guidewire location, with minimal background contamination, during left heart catheterization.

**Conclusions:**

The ability to effectively visualize commercially available X-Ray devices during CMR-guided cardiovascular catheterizations, combined with safe low-SAR pulse sequences, could potentially expedite the clinical translation of interventional CMR.

**Electronic supplementary material:**

The online version of this article (doi:10.1186/s12968-014-0088-7) contains supplementary material, which is available to authorized users.

## Background

Cardiovascular magnetic resonance (CMR) guidance during cardiovascular catheterization procedures offers an ionizing radiation-free alternative to conventional X-Ray guidance [1-3]. This reduced exposure to cancer-causing radiation is particularly desirable for pediatric applications [1,4]. In addition, CMR flow measurements may add speed and fidelity compared with conventional oximetric techniques, and CMR assessment of cardiac function and tissue structure offer value in diagnosis and in guiding therapeutic procedures.

In order to successfully guide a procedure under CMR, we require a very fast frame rate (>5 frames/s) and the ability to modify imaging in real-time [5]. Catheter operators must be able to visualize the tissue, as well as the interventional devices being used. Right heart catheterization has been successfully performed clinically under CMR guidance using catheters with air or gadolinium filled balloons for visualization [6]. Most other applications, such as left heart diagnostic catheterization and catheter interventions, require the use of a rigid guidewire [7] as well as other metallic devices (needles, occluders, stents, etc) [8].

Some commercially available X-Ray devices are composed of paramagnetic materials (eg. nitinol, platinum-iridium), however, inadequate visualization of these devices *in vivo* and concerns of device heating have been limiting [9]. The modest signal void from T2* effects is non-specific and insufficient to safely guide cardiovascular catheterizations. Active devices (with receiver electronics embedded) [10] greatly improve visualization, but have been slow in commercialization and introduction to clinical practice. In addition, new CMR-specific devices often suffer from modified mechanical properties, making mainstream adoption more difficult.

Positive contrast from dephasing gradients has previously been demonstrated for the improved visualization of paramagnetic materials including interventional devices [11-15]. Positive contrast produces bright signal from the paramagnetic material, while suppressing background signal.

In this study, we introduce a high frame rate real-time method for improved visualization of standard commercially available paramagnetic X-Ray devices. We implement a dual echo bSSFP pulse sequence for simultaneous positive contrast device visualization and anatomical imaging during procedural guidance. In addition, we use real-time image processing to depict device signal separately from confounding background signal and we present a color overlay of the device onto anatomical images for improved visualization. *In vivo* proof-of-concept is performed using a commercially available nitinol guidewire for left heart catheterization. If combined with safe, low specific absorption rate (SAR) imaging methods, this visualization method could potentially permit the use of paramagnetic X-Ray devices for CMR-guided catheterizations.

## Methods

### Dual echo bSSFP pulse sequence

The theory of positive contrast using dephasing gradients has been described in detail previously [11]. Briefly, this method exploits the local distortion in the magnetic field caused by the paramagnetic device due to its magnetic susceptibility. A dephasing gradient included in the pulse sequence suppresses background signal because signal is not properly refocused. However, in close proximity to the paramagnetic device, this dephasing gradient is balanced by the local magnetic field gradient caused by the field distortion, such that coherent signal is generated near the device.

**Figure 1:**
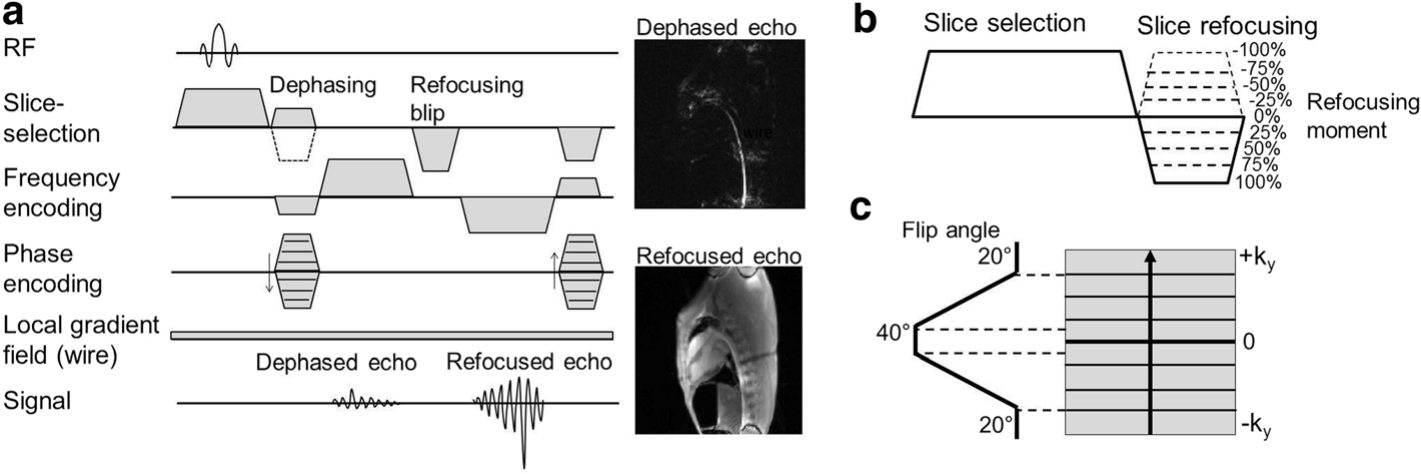
**Dual echo pulse sequence. a)** Dual echo pulse sequence used to generate a positive contrast image (dephased echo) and a standard bSSFP contrast image (refocused echo) simultaneously. Example dephased and refocused images show a nitinol guidewire inserted transfermorally into the descending aorta of a Yorkshire swine. **b)** Variation of the slice refocusing moment as a percentage of the full refocusing moment used for image dephasing. **c)** The variable flip angle scheme used with linear k-space encoding.

A dual echo bSSFP sequence was implemented to generate two types of contrast in the same acquisition (Figure 1a). Dephasing was performed along the slice-selective axis preceding the first echo, creating a positive contrast “device image”. The slice refocusing moment was varied as a percentage (+100% to -100%) of the full slice refocusing moment to generate this dephasing (Figure 1b). A gradient blip in the slice-selective direction was used to complete slice-refocusing for on-resonance spins preceding the second echo to generate an “anatomical image” with standard bSSFP contrast. The zeroth gradient moments were balanced in each TR.

A trapezoidal variable flip angle scheme [16,17] (Figure 1c) was used with linear phase encoding such that high flip angles were used only for the center lines of k-space, in order to reduce the total applied RF energy and reduce SAR.

Partial asymmetric echoes (75%) were used to reduce TR. The dual echo sequence was incorporated into the real-time imaging protocol used for cardiovascular catheterizations. The dephasing moment and variable flip angle were implemented as real-time parameters that can be modified on-the-fly as required by the procedure.

### Image processing

**Figure 2:**
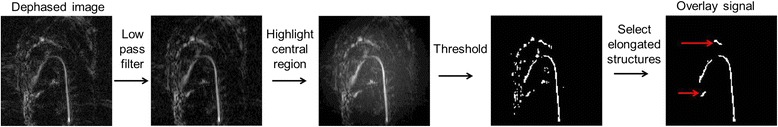
**Image processing method.** Image processing procedure used to isolate guidewire signal from background signal in the dephased image. Images depict a guidewire curving from the aorta into the left ventricle. Red arrows indicate erroneous background signal incorrectly included in the color overlay.

Additional image processing was performed in the Siemens Image Calculation Environment (ICE) in order to detect the device and remove background contamination from other sources of local magnetic field gradients (eg. air-tissue interfaces). A 3-dimensional calibration scan was used to normalize signal intensity variation created by the coil array sensitivity. The following heuristically determined procedure was then applied to the dephased image (Figure 2):

1. Apply Gaussian low pass filter to image
2. Multiply the image by 2D Gaussian in image space to reduce signal from the chest wall relative to the signal in the center of the body.
3. Apply a global signal threshold keeping pixels with [pixel signal > (mean +2 S.D.) image signal]. Simultaneously, apply local adaptive threshold (11 × 11 pixels) to select local high intensity pixels [pixel signal > (mean +1 S.D.) local signal].
4. Connected regions were labeled, and only regions with eccentricity >0.95 (elongated structures) and >12 pixels (large regions) were selected. This step uses prior knowledge that the wire should be a long continuous structure.

Suitable remaining features were overlaid in color on the refocused echo anatomical image and displayed to the operator in real-time (Interactive Front End (IFE), Siemens Medical Solutions, Erlangen, Germany) for procedural guidance.

### Imaging

Imaging was performed on a 1.5 T CMR scanner (Aera, Siemens Medical Solutions, Erlangen, Germany). A 0.035″ Nitrex nitinol guidewire (Covidien, Plymouth, MN) was imaged in a 1 g/L CuSO_4_ phantom. Device position was confirmed with position-matched spin echo imaging (TR = 3 s, TE = 11 ms, FOV = 300 mm, matrix = 192 × 192, slice thickness = 12 mm).

Animal experiments were approved by the institutional animal care and use committee according to contemporary NIH guidelines. Left heart catheterization was performed on three Yorkshire swine using a clinical 0.035″ nitinol guidewire (Nitrex, Covidien, Plymouth, MN) and the dual echo sequence with color overlay for procedural guidance. Four critical locations of the wire tip were considered during the procedure: 1) in the descending aorta, 2) at the aortic arch, 3) at the aortic valve and 4) in the left ventricle. Position-matched X-Ray images were used to validate wire tip position at each of the 4 locations. The accuracy of the color overlay was assessed in each location using 50 frames of continuous scanning of the stationary wire. The number of frames in which the color overlay failed to accurately represent the guidewire tip and the number of frames that contained overlay artifacts which could be mistaken for the wire were determined.

Dual echo imaging parameters were as follows: TE = 0.93 ms and 2.4 ms, TR = 3.37 ms, FOV = 300 mm (phantom) and 350 mm (in vivo), matrix = 192 × 192 fully sampled (phantom) and 192 × 144 GRAPPA acceleration factor 4 (*in vivo*), flip angle = 20° to 40°, refocusing moment = -50%, receiver bandwidth = 1000 Hz/Px, slice thickness = 12 mm.

## Results

### Dual echo bSSFP pulse sequence

**Figure 3:**
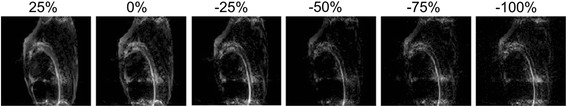
***In vivo*** **positive contrast from a range of dephasing moments.** Modified slice refocusing moments (25% to -100% of full refocusing moment) generating positive contrast signal from the guidewire *in vivo*. A refocusing moment of -50% was chosen for our application.

*In vivo*, positive contrast from the nitinol guidewire was observed for a wide range of refocusing moments <50% (Figure 3). A refocusing moment of -50% was chosen for our *in vivo* applications using this guidewire.

For a refocusing moment of -50%, the dual echo bSSFP required a TR of 3.37 ms, compared to the 2.54 ms of standard real-time bSSFP. Thus, we can achieve a temporal resolution of 175 ms (5.7 frames/second), representing a modest decrease compared to the temporal resolution of the standard bSSFP sequence (132 ms, 7.6 frames/second) (for matrix = 192 × 144, GRAPPA acceleration factor = 4 with 16 reference lines).

The variable flip angle scheme reduced total RF power to 66% of that used for constant 40° flip angle (for matrix 192 × 144, GRAPPA acceleration factor = 4 with 16 reference lines). The point-spread function of the variable flip angle scheme had a full-width at half-maximum of 1.23 pixels, meaning image blurring introduced by this variable flip angle scheme was minimal.

**Figure 4:**
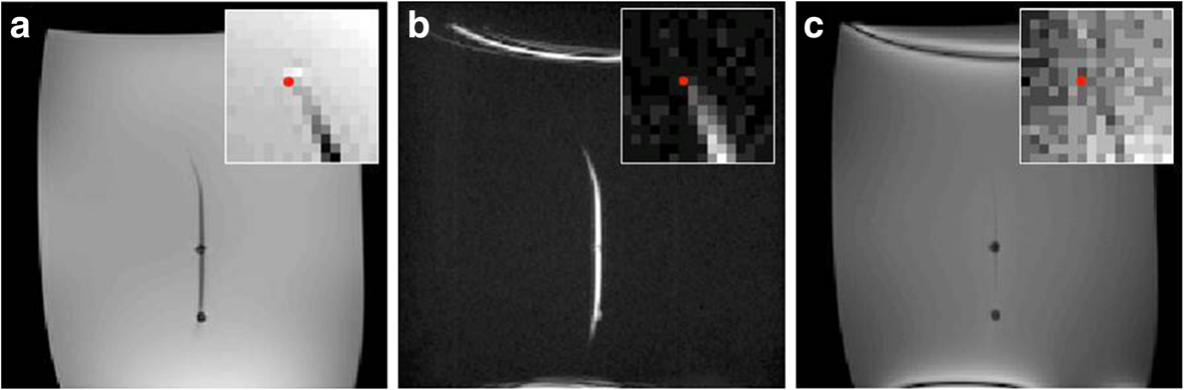
**Images of a guidewire in a phantom comparing spin echo, positive contrast and bSSFP contrast. a)** Spin echo image, **b)** dephased positive contrast image and **c)** refocused bSSFP image of a nitinol guidewire in a CuSO_4_ phantom. A red dot indicating the guidewire tip location determined from the spin echo image is superimposed on all images. The acrylic posts supporting the guidewire in place are also visible **(a, c)**.

**Figure 5:**
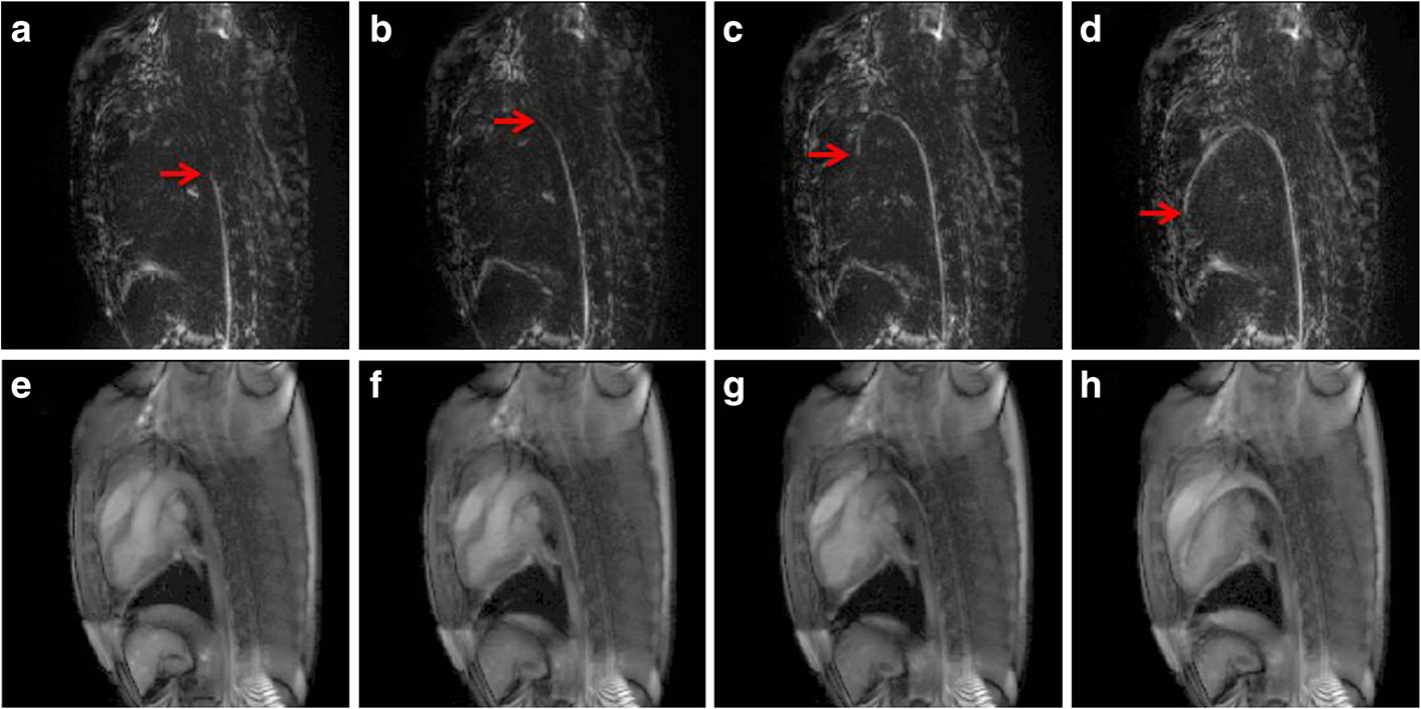
***In vivo*** **left heart catheterization positive contrast and anatomical images from dual echo pulse sequence.** Positive contrast **(a-d)** and anatomical images **(e-h)** generated by the dual echo sequence when the tip is descending aorta **(a, e)**, at the aortic arch **(b, f)**, at the aortic valve **(c, g)** and in the left ventricle **(d, h)**. Red arrows are used to indicate the wire tip.

Comparison of the positive contrast images to spin echo images in a phantom illustrates the clear depiction of the entire length of the wire, including the all-important wire tip (Figure 4). Example *in vivo* images generated by the dual echo sequence at each of the 4 critical locations during left heart catheterization are shown in Figure 5.

### Image processing

All image processing was completed in real-time such that the display was synchronized with image acquisition. Table 1 presents the accuracy of the color overlay in each critical position. The overlay was most unreliable when the tip of the wire was at the level of the valve due to background signal limiting tip detection and motion of the wire tip out-of-plane. Overlay artifacts that could be mistaken for the wire were most prominent when the wire was in the descending aorta.

**Table 1:** **Color overlay assessment during left heart catheterization**

	**Descending aorta**	**Aortic arch**	**Aortic valve**	**Left ventricle**
a) Tip location accuracy	94 ± 2%	86 ± 10%	61 ± 12%	78 ± 11%
b) Deceptive overlay artifacts	21 ± 9%	9 ± 4%	12 ± 7%	9 ± 6%

Using 50 continuously acquired frames of the guidewire stationary with the tip in the descending aorta, at the aortic arch, at the aortic valve and in the left ventricle, the percentage of frames that accurately identify the tip location (a) and the percentage of frames with deceptive overlay artifacts that could be mistaken for the wire (b) are assessed. Mean ± standard deviation from 3 animals is presented.

### Left heart catheterization

**Figure 6:**
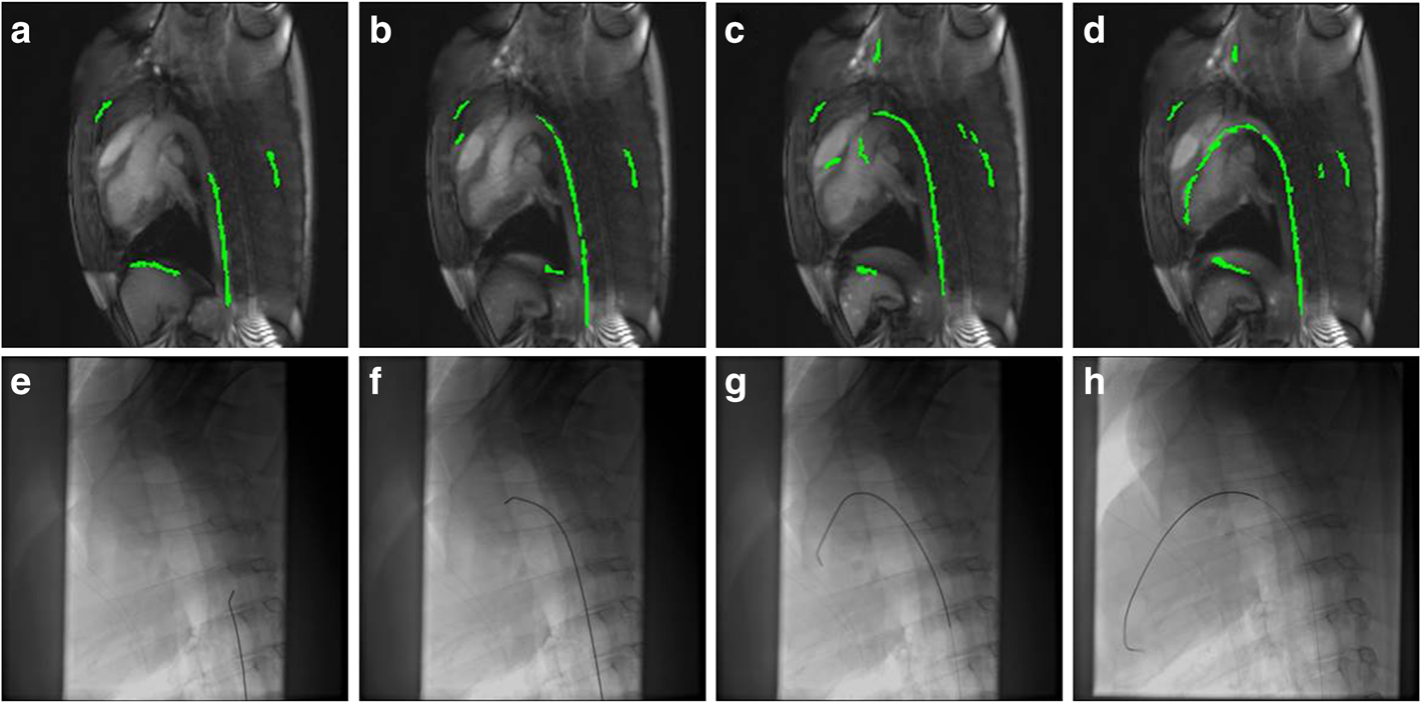
**Left heart catheterization images using dual echo CMR guidance and X-Ray guidance.** Example images during left heart catheterization under CMR-guidance **(a-d)** and X-Ray guidance **(e-h)**. Images are taken at the identical location indicated by markings on the guidewire when the tip is in the descending aorta **(a, e)**, at the aortic arch **(b, f)**, at the aortic valve **(c, g)** and in the left ventricle **(d, h)**.

A movie depicting the IFE visualization during left heart catheterization (see Additional file 1) demonstrates the procedural guidance achieved as the wire moves from the aorta into the left ventricle using a temporal resolution of 175 ms/frame with GRAPPA factor 4. The guidewire visualization afforded by the color overlay shows excellent correspondence with X-Ray at each of the four critical locations (Figure 6). Overall, operators found the visualization easy to use, with most unreliability around the aortic arch. During real-time procedural guidance, dynamic guidewire monitoring (knowledge of wire position and trajectory in previous frames) and tactile feedback assist the operator in compensating for inaccuracies in the color overlay.

## Discussion

### Dual echo bSSFP pulse sequence

Imaging with positive contrast (bright signal) improves visualization of paramagnetic catheter devices compared with traditional signal-void (dark signal) imaging. Here, the extension of previous work to generate a real-time sequence with color overlay represents a large step forward in the use of positive contrast for procedural guidance. The dual echo technique is able to generate two images with different contrasts within the same pulse sequence: a positive contrast image and a traditional bSSFP contrast image. This method provides an efficient use of both time and RF energy. An additional 0.83 ms per TR is required to include the second echo, resulting in only a modest decrease in achievable frame rate. A frame rate of 5.7 frames/second is easily achievable using this sequence with GRAPPA factor 4.

Positive contrast has been used a number of times before for both device visualization and for superparamagnetic iron oxide nanoparticle labeled cell tracking [11-15,18-21]. Previous implementations of positive contrast have used lower flip angle acquisitions [12,18] to exploit off-resonance properties of the bSSFP sequence. For the dual echo sequence, a higher flip angle was used in order to maintain contrast in the anatomical image. Improvements in the positive contrast signal by variations of TR or TE has been demonstrated previously [13,14], however due to the real-time nature of this application, we have chosen to keep these parameters minimized. Other implementations of this method have also used a dephasing gradient in the frequency encoding direction [14], rather than the slice-select direction. Previous *in vivo* applications of positive contrast techniques have also contained background signal from other sources of susceptibility [11,20] and, to our knowledge, ours is the first attempt to isolate the desired signal from the background for a real-time application.

Only modest bSSFP banding artifacts were observed by increasing the TR to 3.37 ms at 1.5 T, which did not degrade the image quality. GRAPPA parallel imaging was used to increase the frame rate. The dephased images contain sparse signal regions compared to the anatomical images and while that causes the dephased images to have less signal overall, the parallel imaging reconstruction actually performs well since there fewer signal bearing regions that alias on to other signal bearing regions in the dephased images. The bipolar readout strategy implemented with the dual echo bSSFP was time-efficient. In our case, no shifts between odd and even echo images caused by off-resonant spins were observed. However, if alternative materials or pulse sequences with a lower receiver bandwidth are used, off-resonance reconstruction may be required to shift the device image into alignment with the anatomical image.

Positive contrast was observed for a wide range of refocusing moments <50% (Figure 3), indicating that one does not have to be too precise in the choice of the dephasing moment to generate positive contrast from the wire. As the dephasing moment is varied, the exact location of signal refocusing in the field distortion close to the wire will vary. When insufficient dephasing is applied, the positive contrast from large anatomical features in the background dominates the dephased image. When excessive dephasing is used, the signal from the wire, especially at the tapered tip, is reduced. A refocusing moment of -50% was chosen for this application with this guidewire. Since the deaphsing moment is specified as a percentage of the required slice refocusing moment, the dephasing parameter does not have to be modified with each change in slice thickness or orientation, however, the real-time control of the dephasing moment allows on-the-fly adjustments for improved performance.

Here we used a bare wire during our *in vivo* experiments. Placing the wire inside of a catheter will change the exact magnetic field distortion. Nevertheless, positive contrast is produced from the guidewire and this technique can be used without modification to visualize the guidewire and catheter combination. The color overlay presented here mimics the appearance of active device visualization [10], which is very useful to interventionists. The nitinol core of the wire tapers at the tip, reducing its positive contrast signal. Around the aortic arch, where there are a lot of tissue boundaries and off-resonance spins, the wire depiction was less accurate (eg. Figure 5c).

As a result of the image processing method which selects elongated structures, overlay artifacts typical manifest as lines outside of the vasculature, which are easily ignored by the operator. Inaccuracies in the overlay are easily compensated in the human mind. In the context of a procedure, the operator has a good idea of the wire tip location even for frames with inaccurate overlay, based on knowledge of the wire trajectory in the previous few frames, as well as tactile feedback.

### Limitations

One limitation of this study is that heating has not been assessed during imaging. Safety studies will be required prior to clinical translation of this method. The variable flip angle technique implemented here will reduce RF power, and thus heating, to 66% compared to the high flip angle (40°) sequence. Temperature increases of 17–48°C have been reported previously under worst-case-scenario conditions with a guidewire in a phantom [22-24], but blood flow *in vivo* helps to limit heating [10]. Alternate methods to reduce heating include moving to lower flip angle gradient echo sequences and RF-efficient non-Cartesian k-space trajectories, still generating positive contrast with dephasing gradients. We speculate that these RF-efficient sequences will reduce the heating to a reasonable level and will investigate heating in future studies.

In some frames, the image processing failed to accurately depict the device, particularly around the aortic arch. Future work is required to improve the specificity of device detection by moving to gradient echo imaging, or adding a third echo to isolate susceptibility sources [25]. In addition, around the aortic arch and in the left ventricle, the wire tip can move out-of-slice with cardiac motion and blood flow, which is an inherent limitation to CMR-guidance with passive devices. Thick slices up to approximately 30 mm generate positive contrast from the guidewire and can be used to locate the guidewire and choose appropriate slice positions. The implementation of intelligent slice tracking [26] using thick slices would improve the clinical utility of this method.

### Translation to other devices and procedures

Proof-of-concept work was done here using a guidewire, however these techniques should be easily translated to other commercially-available paramagnetic devices (eg. needles, stents, cardiac and vascular occluders) as well as to novel CMR-specific passive devices using paramagnetic materials. For each application, the pulse sequence will remain the same, but the image processing may change. Specifically, the appropriate feature detection would need to be assessed. Left heart catheterization was chosen as a straightforward proof-of-concept procedure, but this work could pave the way to more complicated CMR-guided catheter-based procedures using off-the-shelf guidewires.

## Conclusions

We have demonstrated a dual echo positive contrast technique for real-time visualization of commercially available passive paramagnetic devices during cardiovascular catheterizations. Image processing was used to isolate the device signal and generate a color overlay of the device onto the anatomy instantaneously. Proof-of-concept was performed using an off-the-shelf nitinol guidewire for left heart catheterization in swine. Future work will extend the application of this technique to other devices. Good visualization of standard commercially available devices, in combination with safe low-SAR sequences, could permit interventionists to use commercially available paramagnetic X-Ray devices effectively for CMR-guided catheterizations.

## Additional file

Additional file 1:
**CMR-guided left heart catheterization video.** CMR guided left heart catheterization in a swine using a commercially available nitinol guidewire visualized with the real-time dual echo pulse sequence with color overlay. Real-time imaging and image processing generates a temporal resolution of 175 ms/frames (with GRAPPA factor 4).

## Abbreviations

2D: Two-dimensional
bSSFP: Balanced steady state free precession
FOV: Field of view
GRAPPA: Generalized autocalibrating partially parallel acquisition
ICE: Image calculation environment
IFE: Interactive front end
RF: Radiofrequency
SAR: Specific absorption rate
SD: Standard deviation
TE: Echo time
TR: Repetition time
